# United States Circuit Court—The Dental Rubber Cases

**Published:** 1874-01

**Authors:** 


### UNITED STATES CIRCUIT COURT.

### "THE DENTAL RUBBER CASES—A NOVEL LAW SUIT."

"The Goodyear Dental Vulcanite Company, owners of the patent for hard-rubber plates for artificial teeth, have just instituted a suit in the United States Circuit Court, in this city, against Samuel S. White of Philadelphia, laying their damages at $100,000; the alleged offence complained of being what is known in law as "maintenance," which is defined in the books as "intermeddling in a suit that in no way belongs to one, by maintaining or assisting either party with money or otherwise to prosecute or defend it."

Mr. White is an extensive manufacturer of materials which are purchased by dentists for their practice. The dentists are opposed to the payment of royalties to the company, which owns the patent, and it is alleged that Mr. White prepared defences which, it is claimed, he supposes the dentists might make against the company when called on to pay for their licenses; that he voluntarily furnished at his own expense such defences, in a printed form, to the licensees of the company and to others; and that he paid the defendants' expenses of lawsuits instituted by the company against those who have followed his advice. The company claim that their losses by the alleged interference of Mr. White are not less than $100,000.

The other side remains to be heard, and when Mr. White's answer has been put in it will be duly noted."

The above, from the *New York Herald* of Dec. 16, indicates that the Rubber Company is beginning to realize that there is somebody astir—they are alarmed. Well, who would not be, at the prospect of the cutting off of a hundred thousand dollars?

This is merely a poor apology for a *bluff*, and a free admission that they have been *check-mated*; it is an evidence of *despair*, and that the dying struggle is rapidly approaching.

The profession have such entire confidence in the successful issue of the contest now in hand, that they decline to enter into any further negotiations with the Rubber Company, till the rights they claim are fully established.

We will doubtless hear from Mr. White on this matter, in the January issue of the *Cosmos.*

